# Bromodomain and extra-terminal proteins in solid tumors: regulators of immune microenvironment and emerging therapeutic targets

**DOI:** 10.3389/fimmu.2025.1727365

**Published:** 2026-01-09

**Authors:** Deeksha Sharma, Grace G. Bushnell, Alexander P. Kalman, Chloe M. Hutchens, Monika L. Burness

**Affiliations:** 1 Rogel Cancer Center, University of Michigan, Ann Arbor, MI, United States; 2 Biomedical Engineering, University of Minnesota, Minneapolis, MN, United States

**Keywords:** B cells, BET inhibitors (BETi), bromodomain and extra-terminal proteins (BET proteins), NK cells, T cells, tumor-associated macrophages (TAMs)

## Abstract

Bromodomain and Extra-Terminal domain (BET) proteins are key epigenetic readers that recognize and bind acetylated lysine residues on histones, orchestrating transcriptional programs that drive oncogenic processes. BET proteins regulate the expression of oncogenes involved in proliferation, survival, and differentiation, thereby promoting tumor initiation, progression, and therapy resistance across a wide range of solid tumors. Recent findings implicate BET proteins in maintaining cancer stem cells (CSCs), a subpopulation of tumor cells characterized with self-renewal capacity, plasticity, and the ability to evade conventional therapies. In CSCs, BET proteins coordinate stemness-associated transcriptional networks, and drive tumor persistence, metastasis, and relapse following treatment. BET proteins also shape the tumor immune microenvironment by modulating the expression of key immune checkpoint molecules such as PD-L1, regulating cytokine production, and controlling antigen presentation, which collectively influence adaptive and innate immune responses. BET inhibition enhances T cell infiltration and activation while suppressing the immunosuppressive functions of tumor-associated macrophages. The dual role of BET proteins in controlling both stemness and immune regulation positions them as central regulators of tumor-intrinsic and immune-mediated mechanisms in cancer. This makes BET proteins attractive therapeutic targets, as their inhibition offers the potential to simultaneously suppress tumor growth and reprogram the immune microenvironment. Preclinical and early clinical studies demonstrate that combining BET inhibitors with chemotherapy, targeted therapies, or immune checkpoint blockade synergizes anti-tumor responses. Future research focused on understanding the context-specific functions of BET proteins, and optimizing combination strategies will be critical to fully harness their therapeutic potential in solid tumors.

## Introduction

Bromodomain and extraterminal domain (BET) family of proteins comprising BRD2, BRD3, BRD4, and BRDT function as epigenetic readers that recognize acetylated histones, promoting chromatin accessibility (1). By binding to acetylation marks, BET proteins recruit co-activators and transcriptional machinery to promoter and enhancer regions, facilitating assembly of the pre-initiation complex (2, 3). Additionally, BET proteins support transcriptional elongation by stabilizing RNA polymerase II and interacting with elongation factors (1–4). The molecular biology of BET proteins has been comprehensively reviewed (3).

Although BET proteins share conserved bromodomains, individual BET proteins exhibit non-redundant roles (5). BRD4, the most extensively studied BET protein, recruits P-TEFb to promoters and super-enhancers to drive transcriptional elongation of oncogenes and also contributes to immune evasion (2, 6, 7). BRD2 acts as a scaffold for E2F transcription factors and chromatin-modifying enzymes and can promote therapy resistance through activation of the Ras/ERK pathway (8–10). BRD3 exhibits context-dependent activity: in some tumors it promotes metastasis and tumor progression, whereas in others it has tumor-suppressive effects, positively correlating with p21 expression (11–16). While all BET proteins are broadly expressed in normal tissues, BRDT is predominantly expressed in the testis and ovary (1) with its activity largely confined to spermatogenesis, where it coordinates the chromatin remodeling essential for sperm maturation (17). Functional specificity in BET proteins is largely dictated by the C-terminal, extra-terminal, and coiled-coil domains rather than the bromodomains themselves (18).

In cancer, BET proteins are often overexpressed and contribute to oncogenesis by promoting the transcription of proto-oncogenes (19, 20). Recent findings underscore their role in regulating cancer stem cells (CSCs), a subpopulation of tumor cells with self-renewal potential, therapeutic resistance, and metastatic capacity (21–23). Beyond their tumor-intrinsic effects, BET proteins also modulate the tumor microenvironment (TME) by regulating immune cell function and phenotype.

Two major therapeutic approaches have been developed to target BET proteins: inhibitors, which block bromodomain–acetyl-lysine interactions, and degraders, which use proteolysis-targeting chimeras (PROTACs) to induce selective protein degradation (24, 25). Both BET inhibitors (BETi) and BET degraders (BETd) reduce CSC viability, suppress self-renewal, and enhance chemosensitivity in preclinical models of breast cancer, squamous cell carcinoma, and other tumor types (21, 22, 26–28). While pan-BET inhibitors inhibit BRD2, BRD3, and BRD4, efforts are increasingly focused on inhibitors with paralog- or isoform-specificity. Selective inhibition has differential effects: BRD4 inhibition alone suppresses tumor growth in multiple myeloma, AML, and solid tumors, whereas dual BRD2/BRD4 inhibition is often required in resistant models (6, 29). Emerging BET degraders and PROTACs allow selective targeting of BRD2, BRD3, and BRD4, enabling dissection of these non-redundant roles (30, 31). The development of BRD4-selective inhibitors and ET-domain-targeting molecules offers a promising strategy to enhance therapeutic potency while reducing off-target effects (18, 31–38). Together, these strategies enhance the precision of BET targeting; yet a recent multi-tumor analysis demonstrated that adaptive BRD2 overexpression can sustain transcriptional programs and drive resistance, emphasizing the need to integrate combination or sequential therapies into future treatment approaches (39).

Preclinical studies demonstrate that BET-targeted therapies can reprogram tumor-associated macrophages (TAMs), enhance antigen presentation by dendritic cells, boost cytotoxic activity of T and NK cells, and promote anti-tumor immunity by reducing immunosuppressive cytokines (40, 41). However, these therapies can also negatively affect certain immune subsets, underscoring the need to balance efficacy with immune safety (42, 43). BET inhibitors have advanced to clinical development as promising therapies, with potential synergy with chemotherapy and immunotherapy (44).

This review focuses on BET protein regulation of immune cells in the tumor immune microenvironment, a dynamic ecosystem that engages in bidirectional crosstalk with tumor cells and can exert both pro and anti-tumorigenic effects. BET proteins influence immune evasion, inflammatory signaling, and responsiveness to immunotherapy by modulating immune cell function. Understanding how BET-targeted therapies reprogram the immune cells and identifying the conditions under which they potentiate or suppress immune function, are pivotal for optimizing BET-targeted therapies into clinical practice.

## BET proteins in cancer and immune regulation: context and lineage dependency

BET proteins are central transcriptional regulators whose specific roles vary depending on cancer lineage, tumor subtype, and cellular context. This heterogeneity among different cancer types is a result of distinct transcription factor activity, and chromatin and enhancer landscapes across different cancers. BET proteins cooperate with oncogenic drivers that are cancer type specific, including androgen receptor signaling in prostate cancer (45), GLI1 in glioblastoma (46), and MYC activity in hematologic malignancies (47). Even within tumor types, the function of BET proteins can vary; within breast cancer, for example, subtype-specific functions have been described. In estrogen receptor positive tumors, BRD4 cooperates with the estrogen receptor and super-enhancer structures to sustain estrogen-responsive transcriptional programs (48), whereas in triple negative breast cancer (TNBC), BET proteins primarily regulate inflammatory signaling, stemness, EMT, and immune-modulatory axes (22). Recent findings underscore their role in regulating CSCs, a subpopulation of tumor cells with self-renewal potential, therapeutic resistance, and metastatic capacity (9, 22). A comprehensive analysis of bromodomain-containing proteins across cancer types reinforces this heterogeneity, showing that distinct BET genes are differentially expressed in specific tumor lineages (49).This lineage- and cell state-dependence requires a mechanistic and context-specific understanding of BET protein function for successful BET-targeted therapies in the clinic (6).

Beyond tumor-intrinsic effects, BET proteins regulate multiple immune lineages. BRD2 enhances NK-cell cytokine production and cytotoxicity, whereas BRD4 drives inhibitory gene expression via the SMAD4–BRD4 axis (50, 51). In macrophages, BRD4 promotes M2-like polarization through MAF and NRF2/HO-1 signaling, while BRD2 supports pro-inflammatory transcriptional programs (52–54). Neutrophil differentiation is influenced by Znf687-mediated recruitment of a BRD4–SMRT complex to gfi1aa, a transcription factor essential for granulocytic specification (55).

Within the adaptive immune system, BET proteins orchestrate effector differentiation and cytokine output. BRD2 primes Th17 differentiation by increasing early chromatin accessibility, whereas BRD4 sustains high-output IL-17 production (56). BRD4 also promotes terminal differentiation of cytotoxic CD8^+^ T cells through Id2 and Cx3cr1 super-enhancers (57). In B cells, BRD4 cooperates with NF-κB to induce IL-10 and drive regulatory phenotypes, while BRD2 supports mitogenic expansion and activation (53, 58).

Together, these tumor- and immune-intrinsic functions position BET proteins as central transcriptional integrators that shape malignant behavior, immune activation, and immunosuppressive programs in a highly context-dependent manner.

## BET proteins as regulators of the tumor immune microenvironment

The tumor microenvironment consists of malignant cells, immune cells, fibroblasts, and endothelial cells, all organized within a complex extracellular matrix. These cells mediate reciprocal, context-dependent interaction**s** that drive tumor progression, modulate immune evasion, and shape therapeutic outcome (59). While immunotherapies have significantly improved outcomes in hematologic malignancies, their efficacy in solid tumors remains limited (60, 61). Tumor cells, especially CSCs, promote immune evasion and therapy resistance by fostering immunosuppressive niches (62, 63). Given that BET proteins are emerging as critical regulators of TME, they represent a crucial opportunity to improve the function of immunotherapy in solid tumors (41, 54, 64–67).

**Figure 1:**
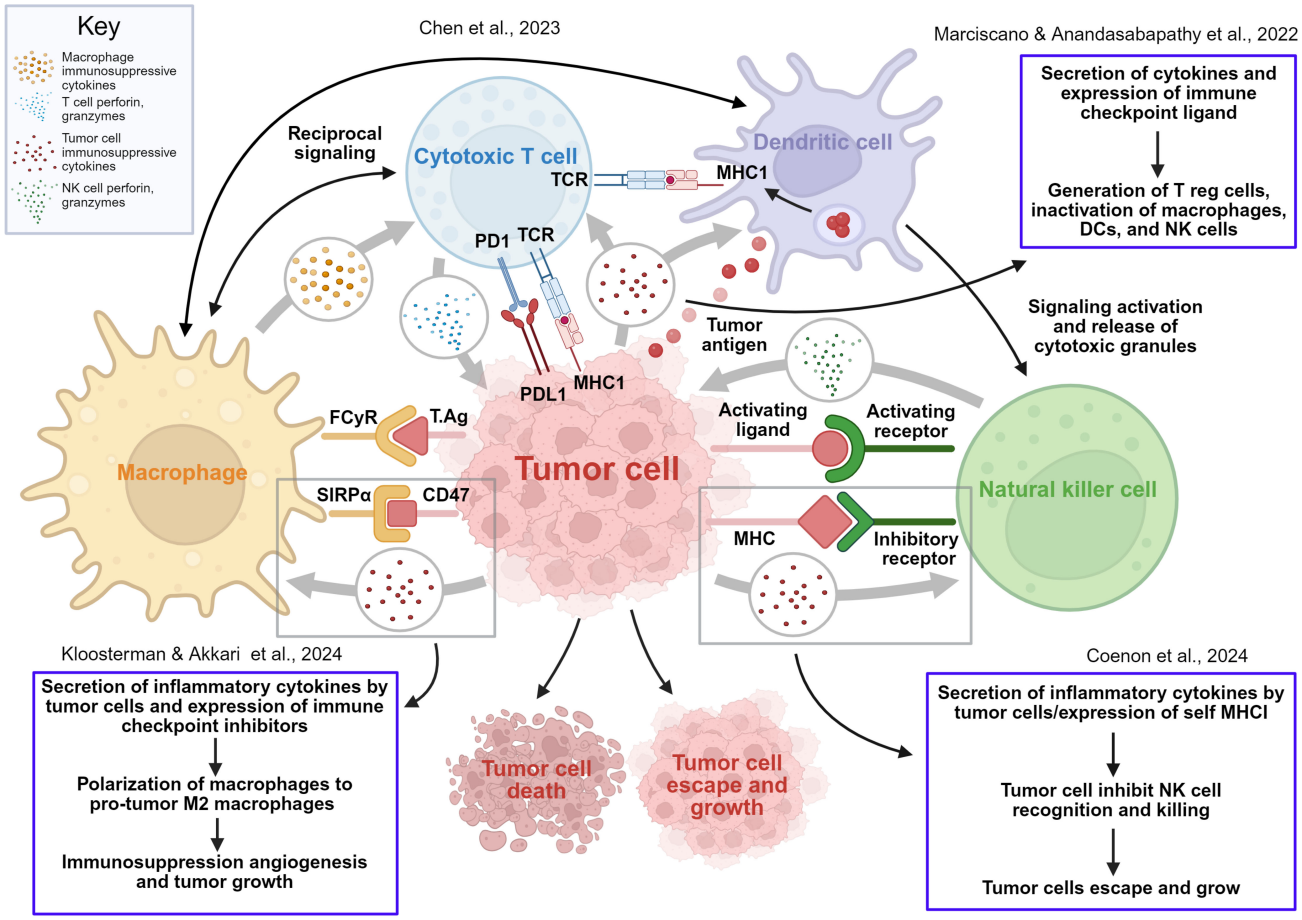
Reciprocal interactions and immunosuppressive signaling among tumor cells and immune cell populations in the tumor microenvironment. This figure comprehensively illustrates the dynamic interplay between tumor cells and key immune cells including Natural Killer (NK) cells, Macrophages, Dendritic cells, and Cytotoxic T cells within the tumor microenvironment, highlighting both effective anti-tumor immune responses and key mechanisms of immune evasion by cancer cells. NK cells, part of the innate immune system, detect and kill tumor cells when their activating receptors bind ligands on the tumor surface, triggering perforin and granzyme release (light green dots) and inducing tumor cell death. Tumor cells can express MHC-I, which binds to inhibitory receptors on NK cells and deactivates them, contributing to immune evasion. Macrophages play dual roles: they can mediate tumor cell killing via phagocytosis and antibody-dependent mechanisms (Tumor antigen (T.Ag) and FcγR interactions) and activate T cells through reciprocal signaling (gold dots). Tumor cells, however, can express CD47, engaging SIRPα on macrophages to deliver a “don’t eat me” signal, preventing phagocytosis and promoting tumor growth. Tumor-derived immunosuppressive cytokines (red dots) can also polarize macrophages from an anti-tumor M1 phenotype to a pro-tumor M2 phenotype, further supporting tumor progression. The adaptive immune response is initiated by DCs, which capture and present tumor antigens via MHC-I to T-cell receptors (TCRs) on Cytotoxic T cells, leading to activation. Activated Cytotoxic T cells specifically kill tumor cells through perforin and granzyme release (light blue dots). DCs also activate NK cells, enhancing the innate response. Tumor cells evade adaptive immunity by expressing PD-L1, which binds PD-1 on Cytotoxic T cells, inhibiting their activity and promoting tumor escape. Overall, this figure shows the complex balance between immune-mediated tumor destruction and cancer immune evasion, providing insights critical for understanding tumor progression and informing immunotherapeutic strategies. Figure is created with BioRender.

As shown in **Figure 1**, immune cells such as cytotoxic T cells, macrophages, dendritic cells, and natural killer (NK) cells use mechanisms including phagocytosis and cytotoxic killing to inhibit tumor growth. However, tumor cells, reshape the immune landscape through oncogenic signaling and the release of immunosuppressive cytokines and chemokines, as well as via cell–cell signaling axes. For instance, tumor-derived cytokines promote macrophage polarization toward a pro-tumor M2 phenotype and suppress phagocytosis through CD47–SIRPα signaling. Additionally, tumor cells evade T cell-mediated cytotoxicity by modulating MHC class I expression and upregulating immune checkpoint ligands such as PD-L1 (68–71).

Preclinical studies suggest that BET inhibitors and BET degraders can reshape the tumor immune microenvironment by suppressing pro-tumor inflammation and restoring cytotoxic T and NK cell activity (40, 41, 72). However, responses are heterogeneous across tumor types, reflecting the complex and context-dependent roles of BET proteins. These context-dependent effects may reflect differences in pre-existing immune composition and myeloid versus lymphoid dominance across tumor types. To better understand these effects, it is essential to examine how BET proteins modulate specific immune populations, including innate and adaptive cells, which collectively determine anti-tumor immunity.

## BET proteins and natural killer cells

Natural killer (NK) cells are key effectors of innate immunity, capable of directly killing tumor cells via perforin- and granzyme-mediated lysis, apoptosis induction, and secretion of cytokines such as CCL3-5, IFN-γ, and TNF-α (73–75). Their cytotoxicity is regulated by a balance of activating and inhibitory receptors, tuned by the local cytokine milieu (76–79). This balance ensures NK cells selective targeting of MHC-I–deficient/low tumor cells while sparing normal cells (80, 81). During tumor progression, NK cell function is often impaired due to upregulation of inhibitory ligands, and immunosuppressive cytokines, and downregulation of activating receptors such as NKp30 and NKp44 (79, 82–85). These changes impair NK cell recognition and contribute to immune evasion, limiting the efficacy of immunotherapeutic strategies that rely on NK cell activation.

**Figure 2:**
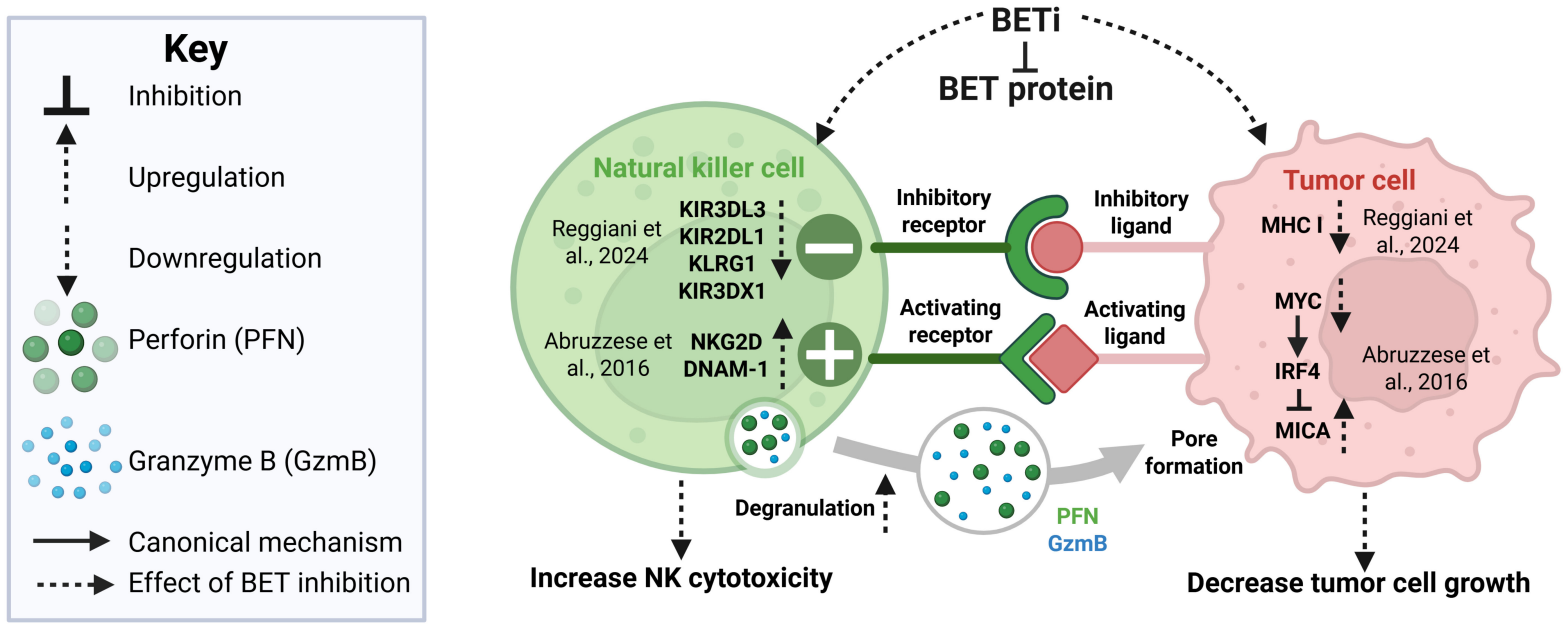
BET inhibition enhances NK-cell–mediated anti-tumor immunity through coordinated effects on NK cells and tumor cells. BET inhibitors (BETi) suppress BET protein activity, reshaping both NK-intrinsic signaling and tumor-cell ligand expression. In NK cells, BET targeting downregulates inhibitory receptors (KIR3DL3, KIR2DL1, KLRG1, KIR3DX1) as reported by Regianni et al. (51), while upregulating activating receptors NKG2D and DNAM-1 (Abruzzese et al., 2016), collectively promoting enhanced degranulation and release of perforin (PFN) and granzyme B (GzmB). In tumor cells, BET inhibition reduces MHC-I and MYC expression (51) and decreases MYC while upregulating the NKG2D ligand MICA (Abruzzese et al., 2016), increasing susceptibility to NK-mediated lysis. Together, these NK-intrinsic and tumor-intrinsic effects strengthen enhance cytotoxicity and reduce tumor growth. Solid and dotted lines represent canonical mechanisms and effects of BET inhibition, respectively. Figure is created with BioRender.

BET proteins influence NK-cell–mediated anti-tumor immunity in a context- and protein-specific manner, affecting both tumor cells and NK-intrinsic functions. For instance, in multiple myeloma, pan-BET inhibition with JQ1(targeting BRD2/BRD3/BRD4/BRDT) increases expression of the NKG2D ligand MICA on tumor cells, enhancing susceptibility to NK-mediated cytotoxicity through a BRD4-dependent mechanism that downregulates c-MYC and alters miR-125b and IRF4 levels (86). In non-small cell lung cancer (NSCLC), BET inhibition with JQ1 and OTX015 disrupts the BRD4-SMAD3 transcriptional axis that drives inhibitory receptor expression on NK cells enhancing NK-cell cytotoxicity and immune-checkpoint molecules (51), whereas in neuroblastoma, JQ1 reduces tumor susceptibility by downregulating ligands for NKG2D and DNAM-1, though the precise mechanisms remain less defined (87). Further, BET inhibitors can synergize with other epigenetic modulators to enhance anti-tumor immunity. In preclinical small-cell lung cancer models, combined BETi (JQ1) and HDAC6 (ACY-1215) produced stronger anti-tumor effects than either agent alone in an NK-dependent manner (40). **Figure 2** illustrates both NK cell–intrinsic and tumor cell–intrinsic BET-regulated pathways identified in studies 52 and 87, and shows how BET inhibitors restores NK cell function, disrupts immune evasion, and supports tumor clearance.

Direct effects of BET proteins on NK cells are also demonstrated: BRD4 is essential for cytotoxicity and IFN−γ production, whereas BRD2 primarily modulates cytokine production without impairing cytotoxicity, and the role of BRD3 remains understudied. This is demonstrated by BET protein–specific responses to inhibition: JQ1, which more strongly targets BRD4, suppresses NK-cell cytotoxicity, whereas AZD5153, with greater selectivity for BRD2, maintains cytotoxic function while modulating cytokine output highlighting distinct roles among BET family members (50).

These findings underscore the context-dependent and multifaceted role of BET proteins in NK cell biology. Depending on the BET family member targeted, the mode of inhibition, and whether effects are direct or mediated via the TME, BETi can either suppress or enhance NK cell responses. Dissecting these differential outcomes shaped by tumor type, immune status, BET protein specificity, and treatment dynamics is essential for the rational design of BET-targeted therapies that harness, rather than hinder, NK cell–mediated anti-tumor immunity. Preclinical studies (**Table 1**) (40, 51, 86) show that BETi, especially in combination with other therapies, can boost NK cell cytotoxicity across tumor models. To optimize efficacy and avoid immune suppression, future strategies should integrate real-time immune profiling, including NK cell subset specific effect and cytokine analysis.

**Table 1:** Preclinical studies examining the impact of BET inhibitors on NK cells and tumor-associated macrophages (TAMs) mediated tumor immunity.

BET inhibitor	Combination	Cancer type	Findings	Reference
(JQ1 and I-BET151) & PROTAC- ARV-825)	N/A (monotherapy)	Multiple myeloma	Enhanced expression of NKG2D activating receptor ligand MICA and tumor sensitivity to NK cytotoxicity via cMYC-IRF4-miR-125b axis	(86)
JQ1 or OTX015	N/A (monotherapy)	Non-small-cell lung cancer (NSCLC)	BETi downregulated NK inhibitory receptors, enhancing NK cell-mediated anti-tumor activity	(51)
JQ1	HDAC6 inhibitor (ricolinostat)	Small cell lung cancer (SCLC)	Combo therapy induced NK cell–dependent synergistic anti-tumor effects and increases MHC II expression in tumor and myeloid cells	(40)
I-BET 762/JQ1	N/A (monotherapy)	Pancreatic	Inhibited pancreatic cancer growth by modulating immune inflammatory c-Myc, p-Erk1/2, IL-6, and p-STAT3	(54)
NHWD-870	N/A (monotherapy)	Breast, ovarian, melanoma & SCLC	Suppressed tumor growth by inhibiting c-MYC and TAM proliferation by inhibiting BRD4-induced CSF1 secretion	(91)
ABBV-075	VEGF antibody (bevacizumab)	Ovarian	BETi induced M2 apoptosis, promoted M1 polarization; combo inhibited tumor growth synergistically and macrophage infiltration, improved survival *in vivo*	(94)
JQ1	ICBi + Hormonal therapy	Prostate	BETi activated STING, non-canonical NF-κB, and type I IFN, induced macrophage- and T cell–dependent immunity	(97)
PLX3397	CSF-1R inhibitor	Melanoma	CSF-1R inhibition enhanced BETi efficacy by targeting CSF-1R^+^ TAMs and reducing PMN-MDSCs	(98)

## BET proteins and tumor-associated macrophages

Tumor-associated macrophages (TAMs) are a heterogeneous population of immune cells. They can polarize into two main phenotypes: M1-like macrophages, which exert anti-tumor effects, and M2-like macrophages, which are pro-tumorigenic. M2-like macrophages promote cancer progression by secreting growth factors, and cytokines that drive epithelial-to-mesenchymal transition (EMT), invasion, metastasis, and therapy resistance (88–94). Given their pro-tumorigenic role, strategies to reprogram M2-like macrophages have attracted significant attention (95).

**Figure 3:**
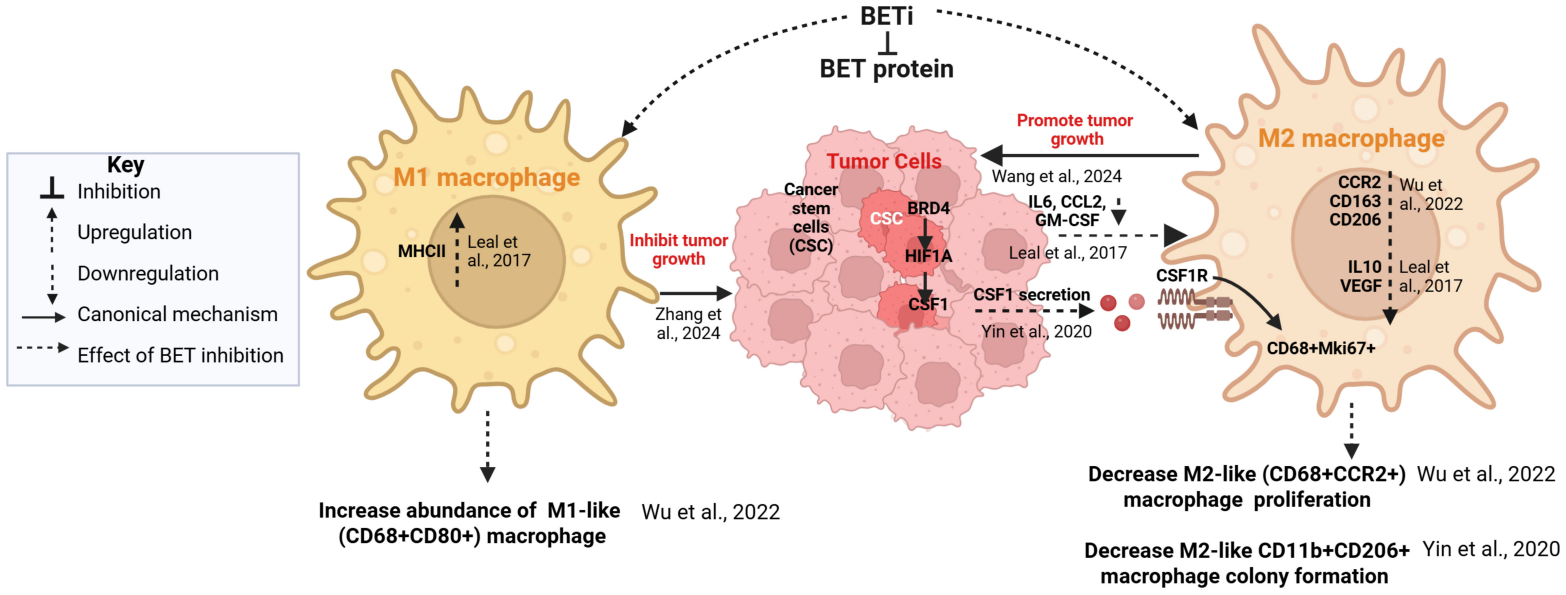
BET inhibition modulates macrophage polarization in the tumor microenvironment. BET inhibitors (BETi) disrupt BET protein function in tumor cells, leading to reduced secretion of pro-tumorigenic cytokines such as IL-6, CCL2, GM-CSF, and CSF1 that drive M2-like macrophage recruitment, proliferation, and expression of immunosuppressive genes. Diminished CSF1–CSF1R signaling limits self-renewal and colony formation of M2-like (CD68+Mki67+) TAMs, while lowered levels of IL-6, CCL2, and GM-CSF inhibit M2 polarization, as demonstrated by decreased expression of CD163, CCR2, CD206, IL-10, and VEGF. Concurrently, BET inhibition promotes the abundance and MHCII expression of anti-tumor M1-like (CD68+CD80+) macrophages. These coordinated effects not only attenuate TAM-mediated promotion of tumor growth, invasion, and immune suppression, but also facilitate enhanced responses to immunotherapy. Solid and dotted lines represent canonical mechanisms and effects of BET inhibition, respectively. Figure is created with BioRender.

BET protein inhibition has emerged as a promising approach to modulate TAM function. BET inhibitors such as JQ1 and I-BET762 suppress M2-like programming, shifting TAMs toward an anti-tumor M1-like phenotype by downregulating immunosuppressive genes and reducing recruitment of M2-like macrophages (5, 54, 94, 96). These phenotypic shifts are associated with reduced tumor growth and improved responses to immunotherapy (**Figure 3**). Key preclinical findings supporting these effects are summarized in **Table 1** (54, 91, 94, 97, 98).

Importantly, individual BET family members have distinct and non-redundant roles in TAMs. BRD4 promotes pro-tumor M2-like polarization by driving MAF transcription, whereas BRD2 contributes predominantly to pro-inflammatory gene expression (52, 53, 99). In pancreatic cancer models, BET inhibition reduces HO-1 expression in macrophages and suppresses tumor-promoting cytokines such as IL-6, CCL2, and GM-CSF, demonstrating a direct link between BET activity and the immunosuppressive tumor microenvironment (99). Beyond polarization, BET inhibitors also impact macrophage self-renewal and survival. JQ1 has been shown to impair GM-CSF-driven peritoneal macrophage self-renewal and IL-4-induced alternative (M2-like) polarization (100), highlighting their ability to simultaneously modulate macrophage proliferation and pro-tumor function. In ovarian cancer, targeting CCR2^+^ TAMs with BRD4 specific inhibitor (ABBV-075) selectively depletes this pro-tumor subset, overcoming adaptive resistance to anti-VEGF therapy while sparing M1-like macrophages (94).

Despite promising findings, resistance to BET-targeted therapies have been observed, particularly in TNBC, where TAMs sustain tumor growth through IL-6/IL-10–driven STAT3/NF-κB signaling (96). This highlights the need to overcome compensatory mechanisms that allow TAMs to maintain pro-tumor functions despite BET inhibition. Combination therapies may hold the key to addressing this issue. Co-targeting BET proteins along with key pro-tumor pathways, such as IKBKE or STAT3 signaling, has shown potential to disrupt macrophage polarization and inflammatory signaling simultaneously (64, 101). In addition, direct targeting of cytokines such as IL-6, which is already being targeted clinically with agents like tocilizumab, could potentially be explored in combination with BET inhibitor to further suppress tumor-promoting inflammation (102, 103).

Together, these findings emphasize that BET proteins orchestrate TAM phenotype, proliferation, and survival in a context- and protein-specific manner, and that selective targeting of BET proteins, particularly BRD4, along with rational combination strategies, may optimize TAM reprogramming and overcome resistance, highlighting the therapeutic potential of BET inhibitors in modulating the tumor microenvironment.

## BET proteins and neutrophils

Neutrophils are the most abundant innate immune cells and play central roles in host defense and inflammatory responses (104). Within the TME, neutrophils constitute a major infiltrating population (105) and exhibit phenotypic and functional heterogeneity (106–108). Tumor-associated neutrophils (TANs) can adopt anti-tumor (N1) or pro-tumor (N2) states, thereby contributing either to tumor suppression or promotion (104, 109). High TAN levels are generally associated with poor prognosis and reduced therapeutic responses across many cancer types (106–108).

Recent studies have expanded our understanding of BET proteins in neutrophil biology beyond their classical transcriptional roles. In zebrafish hematopoiesis, Znf687 recruits a BRD4–SMRT complex to regulate gfi1aa, a transcription factor essential for neutrophil lineage specification (55). Although studied in development, this mechanism highlights BRD4 role in neutrophil differentiation raising the possibility that BET proteins could shape TAN composition and maturation in tumors. In a Th17-driven murine model of severe refractory asthma, the pan-BET inhibitor CPI-203 reshaped inflammatory cytokine networks, increasing IL-1α, IL-1β, IL-2, IL-6, IL-10, IL-12 isoforms, IL-13, and IL-17A, while decreasing G-CSF, CXCL1, MIP-1β, and CCL5 (110, 111). These results demonstrate that BET inhibition alters cytokine circuits controlling neutrophil trafficking (110). Similarly, in a renal ischemia–reperfusion injury model, BRD4 inhibition dampened neutrophil activation, indicating that BET proteins influence neutrophil effector response in tissue stress (112).

Together, these studies highlight BET proteins as regulators of differentiation of cytokine networks and neutrophil effector functions. Although no studies have directly examined BET proteins in neutrophils in cancer, evidence from developmental and inflammatory models suggests they may influence TAN maturation and functional plasticity (110). Given the critical role of TANs in tumor progression, metastasis, and therapy resistance (113), investigating BET-mediated regulation of neutrophils in the TME represents an important and unexplored area in cancer epigenetics.

## BET proteins and dendritic cells

Dendritic cells (DCs) are professional antigen-presenting cells (APCs) that form a critical link between innate and adaptive immunity. In the TME, DCs sense damage-associated molecular patterns (DAMPs) such as calreticulin, ATP, and HMGB1 released during immunogenic cell death (ICD) (114). This sensing promotes the uptake of tumor antigens and DC maturation. Mature DCs then present tumor antigens via MHC molecules to T cells, driving cytotoxic immune responses critical for effective immunotherapy (115, 116). However, the immunosuppressive TME impairs DC function by skewing them toward tolerogenic phenotypes through tumor-derived cytokines, downregulation of co-stimulatory molecules, and upregulation of immune checkpoint (IC) ligands, ultimately dampening T-cell activation and anti-tumor immunity (117, 118).

BET protein targeting exerts context-dependent effects on DCs, with both direct and indirect mechanisms. In a colorectal cancer model, PROTAC-mediated degradation of BET proteins (BRD2, BRD3, and BRD4) in tumor cells induces immunogenic cell death and activates DR5-mediated apoptosis. This leads to enhanced release of DAMPs that promote DC-mediated phagocytosis and cross-presentation, indirectly boosting DC function (65). In contrast, direct exposure of DCs to pan-BET inhibitors, such as JQ1 or I-BET151, suppresses DC maturation. Under these conditions, LPS-induced upregulation of co-stimulatory molecules (CD80, CD86, CD40) and MHC class II is reduced, resulting in impaired T-cell priming (42, 43, 119). Mechanistically, JQ1 inhibits STAT5 phosphorylation and nuclear translocation in human DCs, which is essential for the expression of maturation markers (119)While previous studies suggests that BRD2 may be the predominant BET family member regulating STAT5 (120) isoform-specific knockdowns have not yet been performed in DCs (120–122) so the contributions of BRD3, BRD4, and BRDT remain unresolved.

These findings highlight the context-dependent effects of BET targeting on DC biology. Future strategies should focus on optimizing dosing regimens and exploring combination therapies that preserve DC maturation while enhancing their activation, ultimately improving BET-based immunotherapeutic outcomes.

## BET proteins and T cells

T lymphocytes (T cells) are a key component of adaptive immunity, responsible for identifying and eliminating malignant cells (123). In the TME, CD8^+^ cytotoxic T cells recognize tumor antigens presented on MHC-I molecules and induce tumor cell death via perforin and granzyme release (124). CD4^+^ helper T cells enhance this response by supporting the activation of cytotoxic T cells, NK cells, and B cells. Conversely, regulatory T cells (Tregs) contribute to immune suppression within tumors, limiting effective anti-tumor responses (123, 125). T cell dysfunction in tumors is driven by the upregulation of immune checkpoints (ICs) and the secretion of suppressive cytokines, both of which contribute to immune suppression in the TME (126–129). Tumor cells overexpress ligands like PD-L1, engaging PD-1 receptors on T cells and leading to exhaustion and loss of cytotoxicity (130–134). Chemotherapy can worsen this by enriching IC-positive cancer cells (135). Additionally, tumors suppress T cell responses by downregulating MHC-I, secreting immunosuppressive cytokines, and expanding Tregs (64, 136–139). While IC inhibitors (ICIs) have improved outcomes for a subset of patients, many tumors remain resistant due to poor immune cell infiltration and the suppressive anti-tumor (132, 133).

BET inhibitors have recently been recognized as modulators of T cell infiltration and anti-tumor immune responses. For example, BET inhibitor I-BET51 enhanced CD8^+^ T cell infiltration and reduced tumor growth in ovarian cancer (72). JQ1 decreased PD-L1 expression through BRD4-dependent occupancy of the CD274 promoter, thereby increasing tumor-associated CD8^+^ T cells producing IFN-γ and granzyme B in lymphoma, prostate, and oral squamous cell carcinoma models (140–142). This underscores a BRD4-specific role in anti-tumor immunity, further supported by adoptive transfer studies, where BRD4 inhibition via the BRD4–p300 axis enhanced BATF expression, improving persistence and anti-tumor activity in adoptive T cell and CAR-T models (143). Similarly, BRD4-specific inhibition (SZU-119) augmented CD8^+^ T cell infiltration, cytotoxicity, and PD-L1 suppression to a greater degree compared with pan-BET inhibitors (144). These studies indicate that BET inhibitor selectivity influences T cell infiltration and cytotoxicity. In addition to selectivity, the therapeutic context such as cancer type or combination therapy further determines anti-tumor efficacy. For example, in hepatocellular carcinoma, JQ1 alone increased PD-L1 via Rab8A, but combining JQ1 with anti-PD-L1 restored cytotoxicity and reduced tumor progression (145). Similarly, enzyme-responsive micellar JQ1 (mJQ1) enhanced tumor suppression and CD8^+^ T cell activation compared with free JQ1 in B16F10 melanoma, particularly when combined with radiotherapy (146). These studies highlight the potential of BET inhibitors in combination immunotherapies.

**Figure 4:**
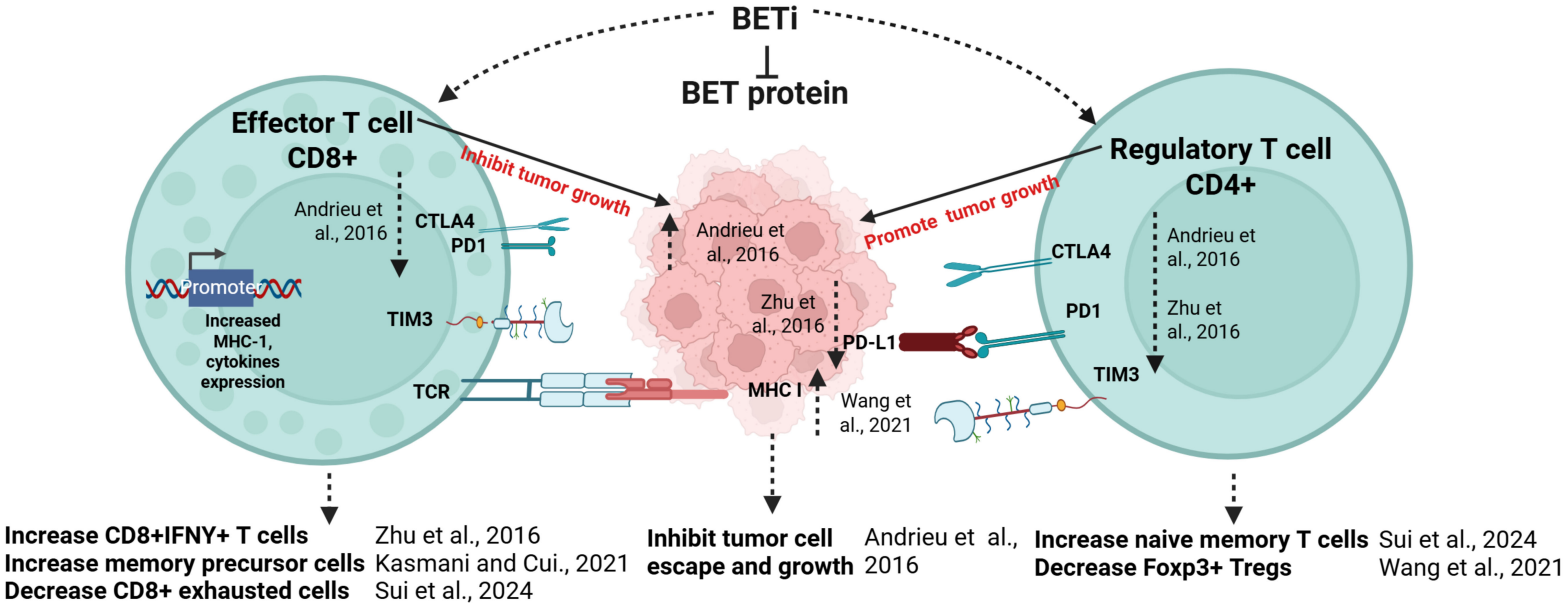
BET inhibition modulates effector and regulatory T cell function in the tumor microenvironment. BET inhibitors (BETi) impact both effector CD8^+^ T cells and regulatory CD4^+^ T cells in the tumor microenvironment, reshaping anti-tumor immune responses. BET inhibitors enhance CD8^+^ T cell activity by increasing IFN-γ^+^ cells and memory precursor populations, while reducing the exhaustion phenotype. BETi also upregulate MHC-I and cytokine expression in tumor cells, diminishing tumor escape and growth. In regulatory T cells, BET inhibition decreases Foxp3^+^ Tregs and increases naïve memory T cells, further alleviating tumor-mediated immunosuppression. Additionally, BETi downregulate inhibitory receptors (PD-1, CTLA-4, TIM-3) and the checkpoint ligand PD-L1, promoting robust anti-tumor T cell responses. Solid and dotted lines indicate canonical mechanisms and effects of BET inhibition, respectively. Figure is created with BioRender.

BET inhibitors can synergize with complementary strategies to enhance anti-tumor T cell responses. They sustain stem-like CD8^+^ T cells and improve persistence and cytotoxicity (143). JQ1 combined with checkpoint blockade (anti-PD-1 or anti-CTLA-4) improved CD8^+^/Treg ratios and Th1 activation in melanoma and lung adenocarcinoma models (147), and similarly enhanced anti-tumor T cell responses in prostate cancer models (141). More recently, BET inhibitors paired with anti-KLRG1 antibodies or IL-2 complexes selectively depleted tumor Tregs and enhanced CD8^+^ cytotoxicity (148). In addition, BET inhibitors (RG6146 and JQ1) sensitize tumor cells to TNF-mediated cytotoxicity by blocking BRD4-dependent NF-κB survival programs in tumor cells. This enhances tumor cell apoptosis in the presence of TNF and, when combined with T-cell bispecific antibodies or IC blockade, increased bystander tumor killing. Overall, BET inhibitors restores T cell function by downregulating IC molecules on T cells and suppressing PD-L1 on tumors, enhancing cytotoxicity and reducing tumor progression (62, 140, 149) as shown in **Figure 4**. Key findings from these models are summarized in **Table 2** (72, 98, 141, 142, 144–146, 148, 150, 151).

**Table 2:** BET protein targeted anti-tumor immunity mediated by T and B cells.

BET inhibitor	Combination	Cancer type	Findings	Reference
I-BET-151	N/A (monotherapy)	Ovarian	Decreased tumor cell viability; increased apoptosis, senescence, and ICD; inhibited metastasis via STAT3; enhanced CD8^+^ infiltration in the TME	(72)
JQ1, RVX208 (BRD2-specific)	anti-CTLA-4 antibody	Prostate	Suppressed PD-L1, increased MHC I and CD8^+^/Treg via antigen processing pathway (additive with anti- CTLA-4)	(141)
JQ1	anti-KLRG1 antibody	Melanoma	Depleted Treg cell populations	(148)
SZU-119 (JQ1+SZU101)	TLR7 agonist (SZU-101)	Melanoma	Increased pro-inflammatory cytokines, decreased PD-L1; increased CD8^+^ T cells and tumor inhibition *in vivo*	(144)
JQ1	N/A (monotherapy)	Malignant melanoma	Suppressed PD-L1 and increased CD8^+^/Treg ratio via IFN-γ upregulation	(146)
JQ1	N/A (monotherapy)	Tongue squamous cell carcinoma	Induced tumor immunogenicity via DAMPs release, promoted DC maturation, increased CD3^+^/CD8^+^ infiltration and reduced tumor growth *in vivo*	(142)
JQ1	PD-L1 blocker	NSCLC	JQ1 and PD-1 synergistically enhanced anti-tumor effect by decreasing Tregs and increasing Th1 cytokines in the TME	(150)
JQ1	PD-L1 blocker	Hepatocellular carcinoma	JQ1 + anti-PD-L1 suppressed HCC by enhancing CD8^+^ T cell activation and cytotoxicity	(145)
PLX51107	N/A (monotherapy)	Melanoma	PLX51107 induced CD8+ T-cell-mediated tumor growth delay by decreasing Cox2, PD-L1, FasL, and IDO-1 expression in the TME	(151)

Although BET-targeted therapies hold significant therapeutic potential, BET proteins play context-dependent roles in T cell biology, with inhibitors showing protein and subset-specific effects. Early studies using pan-BET inhibitors demonstrated that I-BET762 reduces proliferation and cytokine production in CD4^+^ T cells, including IL-17, IFN-γ, and GM-CSF, affecting Th1, Th2, and Th17 subsets (152–154). JQ1 selectively inhibits Th17 differentiation by blocking BRD2/BRD4 binding at IL-17 loci, whereas I-BET762 broadly suppresses Th1 and Th2 cytokines (153). Mechanistic studies showed that BRD2 facilitates early chromatin accessibility to initiate Th17 differentiation, while BRD4 acts as a transcriptional amplifier to sustain high IL-17 production; selective BD1 inhibition with MS402 disrupts Th17 maturation without affecting other subsets (56, 155). Furthermore, BRD4 also drives terminal effector (TE) CD8^+^ T cell differentiation by promoting transcription of Id2 and Cx3cr1 at super-enhancers, whereas loss of BRD4 shifts cells toward memory precursor phenotypes (57). These findings emphasize that BET inhibitor selectivity, dosage, and cellular context critically determine T cell outcomes (153, 154).

To address the complex and context-dependent effects of BET inhibitors on T cell biology, we propose several alternative strategies. For instance, designing selective BET inhibitors that preferentially target BRD4 while sparing BRD2 and BRD3 could help preserve T helper cell function as shown in previous studies (143, 144). In preclinical models, the BET bromodomain inhibitor EP11313 was shown to suppressed pathogenic effector T-cell responses while preserving regulatory T-cell (Treg) function, maintaining IL-10 and TGF-β production (156). These findings highlight the potential to develop BET inhibitors that selectively deplete tumor-associated Tregs while enhancing cytotoxic CD8^+^ T-cell responses. By reshaping the tumor immune microenvironment in this way, such inhibitors could boost anti-tumor immunity and synergize with checkpoint blockade or T-cell–redirecting therapies to improve therapeutic efficacy. Furthermore, optimizing intermittent dosing schedules may allow transient BET inhibition while enabling recovery of normal T cell proliferation and differentiation. Combining BET inhibitors with agents that support T cell activation and survival, such as cytokine therapies (e.g. IL-2, IL-7, IL-21) or co-stimulatory receptor agonists (e.g. OX40, 4-1BB) could enhance cytotoxic responses without increasing T cell dysfunction. Triplet strategies that integrate BET inhibitors with immune checkpoint blockade and either co-stimulatory agonists or Treg-depleting therapies may amplify anti-tumor immunity while maintaining immune balance. Together, these approaches emphasize the need for context-aware and carefully modulated BET targeted application in T cell–based cancer immunotherapy.

## BET proteins and B cells

B cells, a key lymphocyte subtype for humoral immunity, produce antibodies in response to antigen recognition. In cancer immunity, B cells exhibit dual roles based on their phenotype in TME. Effector B cells exert anti-tumor activity through complement activation and antibody production, while regulatory B cells (Bregs) can suppress immune responses by secreting suppressive cytokines and expressing immune checkpoint molecules in response to tumor-derived cytokines (157). Bregs, known for their IL-10 production, have been identified in various cancers (58), and can promote tumor growth by releasing anti-inflammatory mediators and increasing expression of inhibitory molecules such as PD-L1 (158, 159).

Mechanistically, BET proteins are implicated in these regulatory functions. BRD4 associates with NF-κB at the IL-10 promoter in Bregs, and pharmacological inhibition with JQ1 disrupts this interaction, reducing IL-10 production and secretion (58, 158). This suggests that BET inhibitors could potentially counteract the tumor-promoting effects of Bregs. In a similar study, JQ1 treatment reduces B cell class switching, immunoglobulin expression, and antibody secretion (160). The IL-10 suppression appears primarily mediated via BRD4, whereas the reduction in class switching, immunoglobulin expression, and antibody secretion in effector B cells reflects broader BET inhibition. These differing outcomes demonstrate that while BET inhibition can suppress tumor-promoting Breg activity, it may simultaneously impair beneficial effector B cell functions. BRD2, on the other hand, has been linked to general B cell expansion and mitogenesis (161).

While targeting Bregs with BET inhibitors holds therapeutic potential, balancing the effects on effector and regulatory B cell populations will be crucial. The challenge lies in selectively modulating the immunosuppressive functions of Bregs without disrupting the essential antibody-mediated immune responses provided by effector B cells.

## Translational potential of BET targeting in cancer therapy

Preclinical studies establish BET proteins as pivotal regulators of both tumor cells and immune cells within the tumor microenvironment. BET inhibitors effectively modulate transcriptional programs critical for cancer cell survival, proliferation, and immune evasion (3, 24). Collectively, these findings provide a strong rationale for further exploration of BET-targeted therapies across multiple malignancies (19, 162–165). Previous reviews have comprehensively summarized BET inhibitor and PROTAC combination strategies highlighting synergy with chemotherapeutics, targeted therapies, and immunotherapies, and supporting continued preclinical and clinical studies.

Recent preclinical studies demonstrate that BET inhibitors exhibit robust anti-tumor activity as monotherapy across diverse cancers. They induce DNA damage, cell-cycle arrest, and apoptosis while suppressing oncogenic signaling pathways such as IL-6 and BRD4/STRADA/CCND1 (166–169). BRD4-specific inhibitors restore sensitivity to osimertinib in resistant EGFR-mutant lung cancer (170), and induce autophagy-dependent differentiation in glioblastoma (169). Innovative strategies, such as JQ1-loaded nanoplatforms with improved tumor targeting (171) and OPT-0139, a dual BRD4/nitric oxide donors, further enhance therapeutic efficacy (172). Notably, a case report described an exceptional response to BMS-986158 (BETi) in a patient with BRD4-NUTM1 NUT carcinoma harboring a BRD4 splice-site mutation, highlighting the potential for personalized BET-targeted therapy (173). Targeting BRD3 also effectively suppresses nuclear TYRO3-driven metastasis in colorectal cancer, underscoring BRD3 as a promising therapeutic target to prevent tumor dissemination (14).

Preclinical studies demonstrate that BET inhibitors enhance the efficacy of radiation therapy across multiple cancer models. In breast cancer, BRD4 inhibitor combined with radiotherapy reduced PD-L1 and HIF-1α expression, remodeled myeloid cells toward an immunostimulatory phenotype, and expanded CD4^+^/CD8^+^ T cell populations (174). Similarly, BET blockade increases radiosensitivity in glioma and lung cancer by promoting DNA damage and G2/M checkpoint arrest (175), while in glioblastoma it suppresses super-enhancer-driven COL1A1, impairing DNA repair and enhancing tumor cell death (176). BET inhibitors also synergize with targeted radiotherapy, as shown by combination with the PSMA-directed alpha-emitting radioligand [²¹²Pb]Pb-AB001, which further suppresses prostate cancer growth *in vitro* (177). Together, these studies highlight BET inhibition as a versatile radiosensitization strategy acting through both tumor-intrinsic and immune-mediated mechanisms.

BET inhibitors also synergize with targeted therapies to enhance anti-tumor efficacy across diverse cancers. CBX3-targeted dual BET/PLK1 inhibition amplifies the effects of CDK4/6 inhibitors by disrupting BRD4/PLK1-mediated transcription and inducing cell-cycle arrest in prostate cancer (178). In melanoma, BET blockade increases sensitivity to the multi-kinase inhibitor sunitinib by suppressing GDF15, promoting apoptosis, and reducing proliferation (179). Co-inhibition of BET and CDK4/6 further destabilizes BRD4 and impairs homologous recombination in breast cancer (180). Similarly, dual PARP1-BRD4 inhibition enhances DNA damage response and anti-tumor activity in breast cancer, while sequential PARP and BET blockade synergistically suppresses glioblastoma growth (181, 182). Targeting BRD4-mediated YAP1 expression in MEK-resistant melanomas also potentiates trametinib therapy, producing strong anti-tumor synergy (183). Beyond single-target combinations, dual HDAC3/BRD4 inhibitors simultaneously disrupt oncogenic transcription and chromatin regulation, broadly enhancing apoptosis across multiple tumor models (184). Collectively, these studies demonstrate that BET inhibitors can be rationally paired with diverse targeted agents to overcome resistance mechanisms and amplify therapeutic responses.

In addition to targeted therapies, BET inhibitor demonstrate potent synergy with immunotherapeutic strategies, reshaping the tumor immune microenvironment and enhancing anti-tumor immunity (**Tables 1** and **2**). In murine models of small-cell lung cancer, melanoma, and TNBC, BET inhibitor enhances tumor antigen presentation, increases cytotoxic T cell infiltration, and synergizes with PD-1/PD-L1 or CTLA-4 checkpoint blockade (185). In TNBC, combining BET inhibitor with paclitaxel and PD-L1 blockade further remodels the tumor microenvironment, promoting immunogenic and senescent transcriptional programs that enhance therapeutic efficacy (186). Complementing these effects, epigenetic modulation with BET inhibitors paired with a TLR7/8 agonist increases T-cell infiltration and activates innate immune signaling, further suppressing tumor growth (187). Novel combinations such as BET inhibitors with SMAC mimetics (SMACm) in PDAC models synergistically inhibit tumor growth, induce multiple cell death pathways, and remodel the immunosuppressive tumor microenvironment to enhance anti-tumor immunity (188). Innovative strategies also include combining BET inhibitor with the BCG vaccine in melanoma, which reprograms T cells toward an activated state, enhances cytotoxicity, reduces exhaustion, increases intratumoral recruitment, and converts “cold” tumors into “hot” tumors, with efficacy confirmed in humanized PDX models (189).

Triple-combination strategies build on these synergistic effects, simultaneously targeting multiple oncogenic or epigenetic pathways to maximize anti-tumor activity. In malignant peripheral nerve sheath tumors, co-inhibition of MEK, BET, and CDK suppresses tumor growth, outperforming single or dual treatments (190). In bladder cancer, the combination of a BET inhibitor with entinostat and cisplatin enhances DNA damage and apoptotic signaling, producing superior anti-tumor efficacy relative to individual or dual agents (191). These preclinical findings illustrate that rationally designed triple combinations can integrate epigenetic, chemotherapeutic, and signaling-targeted interventions to overcome resistance mechanisms and elicit robust therapeutic responses.

Extensive preclinical data demonstrate the efficacy of BET inhibitors both as monotherapy and in combination with radiation, targeted therapies, and immunotherapies. Early-phase clinical trials have begun translating these findings into patient care. Multiple studies (**Table 3**) are currently evaluating BET modulation in solid tumors, with primary endpoints including efficacy, safety, and immune biomarkers. In hematologic malignancies, a phase I trial of PLX51107 combined with azacitidine in relapsed or refractory myeloid cancers was generally well-tolerated and showed preliminary clinical activity, supporting further investigation of BET inhibitors with hypomethylating agents (192). Similarly, TQB3617 produced a 31% response rate in relapsed/refractory lymphoma, while RO6870810, either as monotherapy in diffuse large B-cell lymphoma or combined with daratumumab, demonstrated manageable safety and early efficacy signals, underscoring the broad potential of BET-targeted therapies across malignancies (193).

**Table 3:** Ongoing clinical trials of BET inhibitors or degraders alone or in combination.

BET inhibitor	Combination	Cancer type	NCT number	Status	Country	Phase
ZEN-3694	N/A (monotherapy)	Colorectal	NCT05803382	Recruiting	USA	1
BMS-986158, BMS-986378	N/A (monotherapy)	Solid tumor, brain	NCT03936465	Completed	USA, Canada	1
ZEN-3694	N/A (monotherapy)	Prostate	NCT02705469	Completed	USA	1
Birabresib (OTX-015)	N/A (monotherapy)	Midline, TNBC, lung, prostate, pancreatic	NCT02259114	Completed	Belgium, Canada, France, Spain, Switzerland	1
Molibresib (GSK525762)	N/A (monotherapy)	Midline	NCT01587703	Completed	USA, Australia, France, Korea, Netherlands, Spain, UK	1
ZEN-3694	epidermal growth factor receptor inhibitor (cetuximab), BRAF inhibitor (encorafenib)	Colorectal	NCT06102902	Recruiting	USA	1
ZEN-3694	PLK1 inhibitor (tuvusertib)	Endometrial, ovarian	NCT05950464	Recruiting	USA	1
ZEN-3694	microtubule inhibitor (nab-paclitaxel), PD-1 inhibitor (pembrolizumab)	Breast, TNBC	NCT05422794	Recruiting	USA	1
ZEN-3694	CDK4/6 inhibitor (abemaciclib)	Breast	NCT05372640	Recruiting	USA	1
ZEN-3694	CTLA-4 inhibitor (ipilimumab), PD-1 inhibitor (nivolumab)	Solid tumor, ovarian	NCT04840589	Recruiting	USA	1
Pelabresib	JAK1/2 inhibitor (ruxolitnib)	Solid tumor	NCT02158858	Active, not recruiting	USA, Belgium, Canada, France, Germany, Italy, Netherlands, Poland, UK	1 & 2
ZEN-3694	androgen receptor inhibitor (enzalutamide)	Prostate	NCT02711956	Completed	USA	1 & 2
ZEN-3694	histone deacetylase inhibitor (entinostat)	Solid tumor, pancreatic	NCT05053971	Recruiting	USA	1 & 2
ZEN-3694	chemotherapy agent (cisplatin), topoisomerase II inhibitor (etoposide)	Midline	NCT05019716	Recruiting	USA	1 & 2
NUV-868 (BRD4-specific)	PARP inhibitor (olaparib), androgen receptor inhibitor (enzalutamide)	Ovarian, pancreatic, prostate, breast, TNBC	NCT05252390	Recruiting	USA, Australia	1 & 2
Molibresib (GSK525762)	N/A (monotherapy)	Solid tumor	NCT01943851	Completed	USA, Australia, Korea, Spain, UK	2
ZEN-3694	androgen receptor inhibitor (enzalutamide), PD-1 inhibitor (pembrolizumab)	Prostate	NCT04471974	Active, not recruiting	USA	2
ZEN-3694	Testosterone, androgen receptor inhibitor (enzalutamide)	Prostate	NCT06922318	Recruiting	USA	2
ZEN-3694	androgen receptor inhibitor (enzalutamide)	Prostate	NCT04986423	Recruiting	USA, China	2
ZEN-3694	N/A (monotherapy)	Lung	NCT05607108	Recruiting	USA	2
ZEN-3694	PARP inhibitor (talazoparib)	Solid tumor	NCT05327010	Recruiting	USA	2
ZEN-3694	PARP inhibitor (talazoparib)	Ovarian, peritoneal, fallopian	NCT05071937	Recruiting	USA	2
Pelabresib	N/A (monotherapy)	Solid tumor	NCT06401356	Recruiting	USA, Belgium, Italy, Netherlands, UK	3

In solid tumors, early clinical evaluation remains limited but promising. AZD5153, a bivalent BRD4 inhibitor, showed dose-dependent pharmacodynamic activity and a partial response in metastatic pancreatic cancer when administered alone or with Olaparib (194). PLX2853, tested in ARID1A-mutated gynecologic cancers and in combination with carboplatin for platinum-resistant ovarian cancer, demonstrated a favorable safety profile, with modest clinical responses, indicating feasibility and supporting exploration of combination strategies with agents targeting compensatory pathways such as PI3K (195). Conversely, a Phase 1b study combining RO6870810 with the PD-L1 inhibitor atezolizumab in ovarian cancer and TNBC revealed increased immune-related toxicity without synergistic benefit, highlighting the need for careful optimization of immunotherapy combinations (196). More recently, BI 894999 was evaluated in advanced solid tumors, diffuse large B-cell lymphoma, and NUT carcinoma, showing target engagement and tolerable safety, though clinical responses were limited, emphasizing the challenges of BET monotherapy and the rationale for continued combination-focused development (197).

Future clinical strategies should focus on optimizing dosing schedules, incorporating biomarker-driven patient selection, and considering sequential rather than concurrent administration to enhance efficacy while minimizing toxicity. Incorporating immunomodulatory agents into combination regimens may mitigate immune toxicity and enhance therapeutic synergy. High-dimensional profiling and advanced preclinical models (e.g., humanized mice, tumor-organoid co-cultures) provide valuable tools to evaluate these strategies and support rational trial design, including identification of immune biomarkers and patient stratification.

## Discussions and perspectives

BET proteins play a critical role in regulating tumor progression and immune modulation within the TME. BET inhibition can disrupt oncogenic transcriptional programs while reprogramming immune dynamics promoting inflammatory macrophage polarization, enhancing NK cell activity, and reshaping T and B cell responses. These multifaceted effects make BET inhibitors promising candidates for cancer immunotherapy, particularly in combination with immune checkpoint blockade (150, 185, 198).

However, a recent clinical study combining BETi with checkpoint inhibitors revealed significant limitations, including immune-related toxicity and limited synergy (196), underscoring the context-dependent nature of BET-mediated immune modulation. Adding to this complexity, BET inhibitors and PROTACs have been shown to suppress IFN-γ production in NK and dendritic cells, alter T cell cytokine profiles, and expand regulatory T cell activity that may compromise anti-tumor immunity. These immune outcomes vary widely depending on the specific immune cell type, differentiation state, and tumor context (58, 153, 161).

To address these challenges, we propose rationally designed dual or triplet combinations to counterbalance BETi-induced immune suppression. Since IFN-γ is critical not only for NK cell cytotoxicity but also for macrophage activation, dendritic cell function, and T cell effector responses (199) combining BETi with immunostimulatory agents may restore essential immune pathways. For example, STING agonists can enhance IFN-γ signaling (200), while IL-2/IL-7 cytokine agonists (201, 202) and co-stimulatory receptor agonists such as OX40 or 4-1BB (203, 204) promote T cell activation. Although these combinations have not yet been tested with BET inhibitors, their complementary mechanisms present promising avenues for future investigation. Furthermore, intermittent or context-specific dosing strategies may attenuate immune exhaustion and preserve effector T cell function.

Future research should focus on elucidating the molecular mechanisms underlying the contradictory immune effects of BET inhibitors. Advanced preclinical models such as humanized mice and tumor-organoid co-cultures are essential to better recapitulate human tumor-immune interactions and predict clinical responses. High-dimensional profiling platforms, including single-cell RNA sequencing and spatial transcriptomics, offer unparalleled resolution to identify the cellular mechanisms of response and resistance. These approaches are particularly powerful for identifying rare immune subsets, mapping cell–cell interactions within the TME and characterizing treatment-induced changes. Such insights will be critical for guiding the rational integration of BET-based therapies into next-generation immuno-oncology strategies (205).

Additionally, defining the distinct immunologic roles of individual BET family members such as BRD2 versus BRD4 in specific immune subsets will be crucial for developing more selective, safer BET-targeted therapies. With a deeper mechanistic understanding of BET proteins’ roles in immune function, and the development of strategic combination approaches, BET inhibitors hold strong potential to become effective and immune-compatible cancer therapies.
